# Single measurement of plasminogen activator inhibitor-1 in sepsis: is it useful for evaluating the severity or prognosis of sepsis?

**DOI:** 10.1186/s40560-017-0259-3

**Published:** 2017-11-13

**Authors:** Kansuke Koyama, Shin Nunomiya

**Affiliations:** Division of Intensive Care, Department of Anesthesiology and Intensive Care Medicine, Jichi Medical University School of Medicine, 3311-1 Yakushiji, Shimotsuke, Tochigi 329-0498 Japan

**Keywords:** Sepsis, Coagulopathy, Plasminogen activator inhibitor-1, Biomarker, Prognosis

Dear Editor,

We read with interest the retrospective analysis by Hoshino and colleagues on prognostic biomarkers in patients with sepsis [1]. These authors evaluated various biomarkers of sepsis and coagulation/fibrinolysis markers in adult patients with sepsis. They showed that plasminogen activator inhibitor-1 (PAI-1) was the only independent predictive marker of 28-day mortality. In the commentary to the study by Hoshino et al., Iba and Thachil described a crucial role of the fibrinolytic system in the pathophysiology of sepsis, where multiple factors such as PAI-1, thrombin-activatable fibrinolysis inhibitor, and protein C participate in modulating the system [2]. They questioned why only PAI-1, but no other coagulation markers, was associated with mortality. They concluded that they recommend against one-point PAI-1 analysis for assessing coagulation/fibrinolytic systems, and were concerned that the findings by Hoshino and colleagues might mislead future studies. We wish to provide an alternative view in this correspondence.

Inflammation and coagulation constitute two host defense systems with a complementary role against infection in sepsis. Depending on the severity of the systemic inflammatory reaction and the degree of involvement of coagulation derangement, sepsis exhibits various patterns of disease or organ dysfunction. In addition, the severity of inflammation and coagulopathy dramatically changes over time, and how these changes occur may affect the patients’ prognosis. Therefore, for precisely evaluating disease severity and progress at the time, what type of pathophysiological mechanism the markers of sepsis reflect and how it usually changes over time in the course of sepsis need to be understood.

In the study by Hoshino et al. [1], the trends of coagulation/fibrinolysis markers over time between survivors and non-survivors in patients with sepsis are similar to the results of previous studies [3–5]. Notably, the time course of PAI-1 is consistent with our previous results, where a marked increase in its peak was observed in non-survivors on the day of admission, and this promptly decreased over a few days [5]. PAI-1 is a marker of inhibitory fibrinolysis and is excessively induced on the surface of endothelial cells by pro-inflammatory cytokines. This rapid increase in PAI-1 with a peak at around the onset of sepsis may suggest that PAI-1 reflects the severity of inflammation and coagulopathy in the early phase of sepsis. It might be, therefore, a reasonable finding that PAI-1 was a strong predictor of mortality at the time of ICU admission in Hoshino et al.’s study [1].

In Hoshino et al.’s study [1], their finding that only PAI-1 was significantly associated with mortality does not deny a relationship of other coagulation/fibrinolysis markers with mortality or with the pathophysiology of sepsis. In their study, logistic regression analysis was conducted for comparison of variables for the association with mortality, not for adjustment of baseline. Other markers, such as thrombin-antithrombin complex (TAT), protein C, and soluble fibrin, were associated with mortality in univariate analysis. Therefore, their results should be interpreted that there was a certain degree of interrelationship or collinearity among these markers.

In our previous study, TAT at admission was independently associated with mortality in multivariate analysis which also included the variables of PAI-1 and protein C activity [5]. Additionally, TAT’s predictive ability of mortality became more accurate on the day after admission. Therefore, the reasons why other coagulation/fibrinolysis markers were not independent variables in Hoshino et al.’s study [1] could be (1) because of less variety in the study cohort due to an inadequate sample size and (2) timing for measurement of these markers. It is generally to be desired that events per variable is 10 or greater in logistic regression analysis; therefore, at least 60 non-survivors would be needed to include six variables used in the multivariate analysis of Hoshino et al.’s study [1].

We agree with Iba and Thachil [2] that comprehensive evaluation is necessary to understand the status of sepsis. Therefore, biomarkers of sepsis or coagulation/fibrinolysis markers should be precisely applied according to whether they are markers of prognosis, markers of disease severity or progression, or markers of pathophysiology (e.g., reflect increased coagulation, insufficient fibrinolysis, or endothelial injury). Additionally, serial measurements of multiple markers are required to follow the dynamic changes in septic conditions, which consist of complex, multiple pathophysiological mechanisms. At the same time, we suggest that one-point measurement of PAI-1 on admission may reflect the degree of the early inflammatory response and thrombotic defense against infection and therefore would be useful for initial, simple assessment of the disease severity in patients with sepsis.

## Abbreviations

PAI-1: Plasminogen activator inhibitor-1
TAT: Thrombin-antithrombin complex

## References

[1] Hoshino K, Kitamura T, Nakamura Y, Irie Y, Matsumoto N, Kawano Y (2017). Usefulness of plasminogen activator inhibitor-1 as a predictive marker of mortality in sepsis. J Intensive Care..

[2] Iba T, Thachil J (2017). Clinical significance of measuring plasminogen activator inhibitor-1 in sepsis. J Intensive Care.

[3] Lorente JA, Garcia-Frade LJ, Landin L, de Pablo R, Torrado C, Renes E (1993). Time course of hemostatic abnormalities in sepsis and its relation to outcome. Chest.

[4] Kinasewitz GT, Yan SB, Basson B, Comp P, Russell JA, Cariou A (2004). Universal changes in biomarkers of coagulation and inflammation occur in patients with severe sepsis, regardless of causative micro-organism. Crit Care.

[5] Koyama K, Madoiwa S, Nunomiya S, Koinuma T, Wada M, Sakata A (2014). Combination of thrombin-antithrombin complex, plasminogen activator inhibitor-1, and protein C activity for early identification of severe coagulopathy in initial phase of sepsis: a prospective observational study. Crit Care.

